# Protein markers and risk of type 2 diabetes and prediabetes: a targeted proteomics approach in the KORA F4/FF4 study

**DOI:** 10.1007/s10654-018-0475-8

**Published:** 2018-12-31

**Authors:** Cornelia Huth, Christine von Toerne, Florian Schederecker, Tonia de las Heras Gala, Christian Herder, Florian Kronenberg, Christa Meisinger, Wolfgang Rathmann, Wolfgang Koenig, Melanie Waldenberger, Michael Roden, Annette Peters, Stefanie M. Hauck, Barbara Thorand

**Affiliations:** 1 Institute of Epidemiology, Helmholtz Zentrum München – German Research Center for Environmental Health (GmbH), Ingolstädter Landstraße 1, 85764 Neuherberg, Germany; 2 German Center for Diabetes Research (DZD), München-Neuherberg, Germany; 3 Research Unit Protein Science, Helmholtz Zentrum München – German Research Center for Environmental Health (GmbH), Neuherberg, Germany; 4 Institute for Clinical Diabetology, German Diabetes Center, Leibniz Center for Diabetes Research at Heinrich Heine University Düsseldorf, Düsseldorf, Germany; 5 Medical Faculty, Heinrich Heine University Düsseldorf, Düsseldorf, Germany; 6 Division of Genetic Epidemiology, Department of Medical Genetics, Molecular and Clinical Pharmacology, Medical University of Innsbruck, Innsbruck, Austria; 7 Chair of Epidemiology, Ludwig-Maximilians-Universität München, UNIKA-T Augsburg, Augsburg, Germany; 8 Institute of Biometrics and Epidemiology, German Diabetes Center, Leibniz Center for Diabetes Research, Heinrich Heine University Düsseldorf, Düsseldorf, Germany; 9 Department of Internal Medicine II – Cardiology, University of Ulm Medical Center, Ulm, Germany; 10 Deutsches Herzzentrum München, Technische Universität München, Munich, Germany; 11 German Centre for Cardiovascular Research (DZHK), Partner Site Munich Heart Alliance, Munich, Germany; 12 Research Unit of Molecular Epidemiology, Helmholtz Zentrum München – German Research Center for Environmental Health (GmbH), Neuherberg, Germany; 13 Division of Endocrinology and Diabetology, Medical Faculty, Heinrich Heine University Düsseldorf, Düsseldorf, Germany

**Keywords:** Type 2 diabetes, Prediabetes, Population-based, Biomarker, Proteomics, Prediction

## Abstract

The objective of the present study was to identify proteins that contribute to pathophysiology and allow prediction of incident type 2 diabetes or incident prediabetes. We quantified 14 candidate proteins using targeted mass spectrometry in plasma samples of the prospective, population-based German KORA F4/FF4 study (6.5-year follow-up). 892 participants aged 42–81 years were selected using a case-cohort design, including 123 persons with incident type 2 diabetes and 255 persons with incident WHO-defined prediabetes. Prospective associations between protein levels and diabetes, prediabetes as well as continuous fasting and 2 h glucose, fasting insulin and insulin resistance were investigated using regression models adjusted for established risk factors. The best predictive panel of proteins on top of a non-invasive risk factor model or on top of HbA1c, age, and sex was selected. Mannan-binding lectin serine peptidase (MASP) levels were positively associated with both incident type 2 diabetes and prediabetes. Adiponectin was inversely associated with incident type 2 diabetes. MASP, adiponectin, apolipoprotein A-IV, apolipoprotein C-II, C-reactive protein, and glycosylphosphatidylinositol specific phospholipase D1 were associated with individual continuous outcomes. The combination of MASP, apolipoprotein E (apoE) and adiponectin improved diabetes prediction on top of both reference models, while prediabetes prediction was improved by MASP plus CRP on top of the HbA1c model. In conclusion, our mass spectrometric approach revealed a novel association of MASP with incident type 2 diabetes and incident prediabetes. In combination, MASP, adiponectin and apoE improved type 2 diabetes prediction beyond non-invasive risk factors or HbA1c, age and sex.

**Electronic supplementary material:**

The online version of this article (10.1007/s10654-018-0475-8) contains supplementary material, which is available to authorized users.

## Introduction

Type 2 diabetes causes an enormous burden for the individual as well as for societies of many countries worldwide [1]. Therefore, improved understanding of disease pathophysiology and the development of preventive measures are particularly important, as well as tools for optimal prediction of future disease occurrence enabling targeted preventive measures. Biomarker data may contribute to these aims [2–5].

Research on type 2 diabetes risk stratification either aims at diagnosis of insulin resistance or prediabetes [6, 7] or attempts to directly predict the future risk of diabetes [7–10]. Ideally, already the development of prediabetes is prevented as not only diabetic patients but also prediabetic persons may suffer from complications caused by hyperglycemia [11]. Yet, only few studies have developed algorithms to predict incident prediabetes [12].

An important issue for new prediction algorithms or devices is the selection of an appropriate benchmark model. Typical decision points are whether the new algorithm shall add benefit on top of or replace mostly questionnaire-based non-invasive tools or other benchmark biomarkers [13]. Often a combination of both is relevant. Type 2 diabetes is usually diagnosed by fasting glucose or HbA1c concentrations [14] and several other blood markers (most prominently insulin) are known to play important roles in the pathophysiology [3, 15]. Therefore, several biomarkers are already available and a deliberate choice is needed.

The German Diabetes Risk Score (GDRS) is a non-invasive tool and currently recommended for type 2 diabetes screening and risk prediction in Germany [16, 17]. Since there is no international consensus regarding prediction algorithms in the field of type 2 diabetes, we used the established risk factors included in the GDRS for benchmarking in our German study. Additionally, we used HbA1c concentrations, which have been proposed to be evaluated in combination with the GDRS for screening and prediction [17]. The main advantage of HbA1c in contrast to glucose is its spontaneous availability, avoiding fasting, which is no longer required in routine clinical practice [18], and avoiding oral glucose tolerance test (OGTT) burden [19].

Our study aimed to identify novel protein associations with incident type 2 diabetes or incident prediabetes in order to further elucidate pathophysiological processes underlying diabetes development. 14 candidate proteins selected based on previous results from a mouse model on type 2 diabetes (5), unpublished shotgun discovery proteomic analyses, and literature mining were quantified by a targeted selection reaction monitoring (SRM) mass spectrometry (MS) approach. In addition to the dichotomous outcomes, we investigated whether baseline plasma protein levels were prospectively associated with the diabetes-related continuous outcomes fasting glucose, OGTT 2-h-glucose, fasting insulin, and insulin resistance. Furthermore, our study aimed to assess whether our protein panel improved the prediction of incident type 2 diabetes or incident prediabetes on top of established risk factors and to select the protein subsets with the best predictive power.

## Methods

### Study population, definitions of incidence outcomes and selection criteria

The Cooperative Health Research in the Region of Augsburg (KORA) F4 (2006–2008) and FF4 studies (2013–2014) are follow-up examinations of the population-representative KORA S4 study (1999–2001), which was conducted in Augsburg (Germany) and two surrounding counties. The study design has been described previously in detail [20].

Previously known type 2 diabetes was defined as self-report that could be validated by the responsible physician or medical chart review, or as current use of glucose-lowering medication. All participants without known diabetes were assigned to receive a standard 75 g oral glucose tolerance test (OGTT). Blood samples were taken without stasis after an overnight fast of at least 8 h and 2 h after glucose solution intake. Normoglycemia (i.e. fasting glucose < 6.1 mmol/l and 2-h-glucose < 7.8 mmol/l), prediabetes (fasting glucose ≥ 6.1 mmol/l but < 7.0 mmol/l, and 2-h-glucose < 7.8 mmol/l [isolated impaired fasting glucose (IFG)] or fasting glucose < 6.1 mmol/l and 2–h-glucose ≥ 7.8 mmol/l but < 11.1 mmol/l [isolated impaired glucose tolerance (IGT)], or both [IFG and IGT]), and newly diagnosed diabetes (fasting glucose ≥ 7.0 mmol/l or 2-h-glucose ≥ 11.1 mmol/l) were defined according to the 1999/2006 WHO criteria [21, 22]. Newly diagnosed and known diabetic participants for whom the diabetes type could not be validated and for whom no contradictory information was given were assumed to have type 2 diabetes. The outcome diabetes included known and newly diagnosed diabetes.

**Fig. 1:**
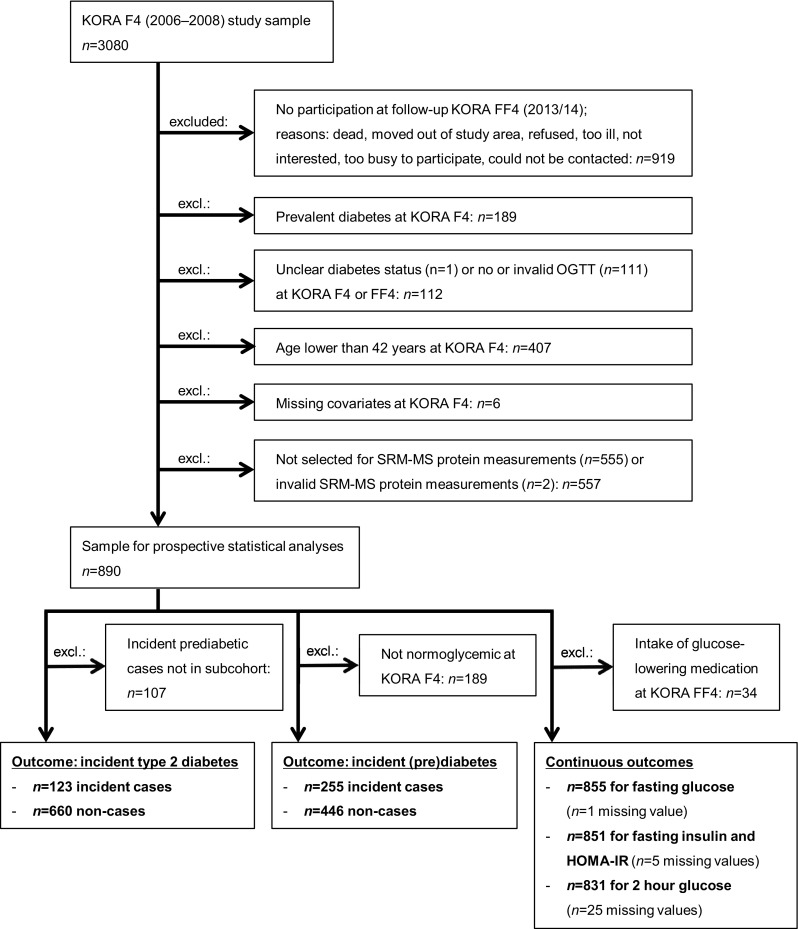
Flowchart showing sample sizes and reasons for exclusions

The KORA F4 study included 3080 participants aged 32–81 years, of whom 2161 also participated in KORA FF4 (Fig. 1). For the current prospective KORA F4/FF4 investigation we excluded 189 KORA F4 participants with prevalent diabetes, 112 participants with unclear diabetes status or missing/invalid OGTT at KORA F4 or FF4, 407 participants younger than 42 years at KORA F4 (due to the low incidence of type 2 diabetes in the lowest 10-year age-category), and six participants with missing covariate data. The remaining 1447 participants qualified for SRM-MS protein measurements. Out of these, we randomly selected a subcohort of 728 participants plus all additional incident type 2 diabetes or incident prediabetes cases. Two participants were excluded due to outliers in the SRM-MS data. The final study comprised 890 participants. The type 2 diabetes analysis sample included 660 non-cases from the subcohort and 123 incident cases. The (pre)diabetes analysis sample contained 446 non-cases from the subcohort and 255 incident cases; these 255 cases comprised 223 prediabetic and 32 diabetic cases who directly progressed from normoglycemia to diabetes. The case-cohort sampling is illustrated in Supplemental Fig. 1. The longitudinal analyses of the continuous diabetes-related outcomes were restricted to 831–855 (depending on outcome) participants with complete data who were not taking glucose-lowering medication.

### Clinical measurements and assessments of risk factors

All participants underwent standard physical and medical examinations at KORA F4 and FF4.

HbA1c in KORA F4 was assessed in hemolyzed whole blood using a cation-exchange high performance liquid chromatographic, photometric assay on an Adams HA 8160 Hemoglobin Analysis System (Arkray Inc., distributed by A. Menarini Diagnostics, Florence, Italy). Insulin levels in KORA F4 were measured in thawed serum by an electrochemiluminescence immunoassay on a Cobas e602 instrument (Roche Diagnostics GmbH, Mannheim, Germany). The assessment of the other metabolic, anthropometric, and lifestyle variables was performed as described [23]. In KORA FF4, glucose concentrations were measured in fresh serum by an enzymatic, colorimetric method using the GLU assay on a Dimension Vista 1500 instrument (Siemens Healthcare Diagnostics Inc., Newark, USA) or using the GLUC3 assay, on a Cobas c702 instrument (Roche). KORA FF4 serum insulin concentrations were assessed by a solid-phase enzyme-labeled chemiluminescent immunometric assay on an Immulite 2000 systems analyzer (Siemens) or by an electrochemiluminescence immunoassay on a Cobas e602 instrument (Roche). The measurement instrument and assays changed in KORA FF4 from Siemens to Roche halfway during the study. Calibration formulas were developed using 122 (194 for insulin) FF4-samples measured with both methods during the time of the change. No calibration was needed for glucose, because the double measurements were very similar. The Siemens insulin results were calibrated to the Roche measurements using the following formula: Insulin_Roche = 7.842 pmol/L + Insulin_Siemens × 1.016. The homeostasis model assessment insulin resistance (HOMA-IR) was calculated as fasting insulin (in pmol/l) × fasting glucose (in mmol/l) ÷ 135.

Parental history of diabetes was defined as positive (at least one parent with diabetes), negative (both parents without diabetes) or unknown diabetes status (else). Sibling history of diabetes was defined as positive (at least one sibling with diabetes) or negative (no siblings or all siblings without diabetes or with unknown diabetes status). Because the KORA F4 participants older than 72 years had not been asked for their parental and sibling history of diabetes, missing values were replaced using the participants’ data from the KORA/MAGiC-Control, KORA S4 or FF4 studies where available. One sibling history value was imputed using covariate data with the package mice in R [24].

### Targeted SRM-MS protein measurements

The plasma samples of 271 persons of the subcohort had already been measured previously (lot 1) [23]. The remaining subcohort and all additional incident cases were newly measured in 2016 when the FF4 data became available (lot 2, *n *= 621). These measurements were performed similarly as published for lot 1 and are described together with the combined data preprocessing in detail in the Supplemental Material.

In short, the 621 lot 2 samples were randomly distributed into thirteen processing batches and quality control was performed as described for lot 1 [23]. All lot 2 sample preparation batches passed quality control. All measured peptides were proteotypic with the exception of the two ‘MASP’ peptides TGVITSPDFPNPYPK and AAGNECPELQPPVHGK which together with the SLPTCLPVCGLPK peptide are transcribed from the *MASP1* gene (according to Ensembl human database, release 90, August 2017). While the ‘SLPT’ peptide only translates into the MASP-1 isoform, the ‘TGVI’ and ‘AAGN’ peptides translate into the protein isoforms MASP-1, MASP-3 and MAP44 [25]. Accordingly, all results for the protein originally termed MASP-1 [23] are addressed in this manuscript as MASP. Isotope-labelled synthetic peptides were used for each peptide as internal control to correct signal integration and for relative quantification as described [23]. Liquid chromatography MSMS analysis was performed on an Ultimate3000-HPLC system (Thermo Fischer Scientific, Dreieich, Germany) coupled online to a QTrap4000 mass-spectrometer (ABSCIEX, Framingham, MS, USA) by a nanospray ion source. This technique measures the area under the curve (AUC) signals of pre-specified collision-induced peptide dissociation products, the so-called transitions.

Coefficients of variation (CV) of pooled samples were calculated for all transition signals based on five replicate measurements per lot using the software AuDIT [26]. Transitions with a CV ≥ 30% were excluded. Light (endogenous) to heavy (synthetic) ratios of the AUC values were calculated, log_2_-transformed, and averaged for all transitions of each peptide. Peptide-level light-to-heavy-ratios were averaged per protein to yield relative protein levels. Within this process, the data was corrected for technical covariates. Quality control of signals was based on CV-results of lot 1 and lot 2 pools and 29 duplicate measurements of lot 2 samples. Only peptides demonstrating reliable reproducibility were included in the combined analysis, leaving a total of 30 peptides in lot 1 and 31 peptides in lot 2, and representing 14 candidate proteins (Supplemental Table 1). The SRM-MS signals of all analyzed transitions are shown in Supplemental Fig. 2 exemplarily for a plasma sample pool. The work-flow from plasma depletion, via SRM-MS measurement, to computation of multivariable adjusted odds ratios (ORs) is illustrated in Supplemental Fig. 3.

### Statistical analysis

Statistical analyses were performed using R version 3.4.2 [27]. All SRM-MS protein-level light-to-heavy-ratios were divided by their sex-specific SDs. Associations between standardized protein light-to-heavy-ratios and incident type 2 diabetes/incident (pre)diabetes were analyzed by logistic regression. For the prospective analyses between protein light-to-heavy-ratios and continuous diabetes-related outcomes, all diabetic participants using glucose-lowering medication were excluded; the glucose and insulin variables were log_e_-transformed and *z*-standardized and analyzed by linear regression, adjusting the follow-up outcome for the respective baseline variable.

The association analyses focusing on pathophysiological mechanisms were adjusted for important baseline type 2 diabetes risk factors, i.e. for sex (male/female), age, waist circumference, and height (all continuous) (model 1), plus smoking (current/former/never), physical inactivity (inactive/active), actual hypertension (yes/no), triglyceride concentration, and total cholesterol/HDL-cholesterol ratio (model 2a). Model 3a was adjusted for all variables available in KORA F4 of the primary prediction reference model, the non-invasive GDRS [16] (GDRS_adapted_, the adaptations are described in the Supplemental Material). Compared to model 2a, the two lipid variables were replaced with parental (both parents/one parent/unknown/no) and sibling (at least one sibling/no sibling) history of diabetes. The models 2b and 3b were additionally adjusted for baseline HbA1c concentrations.

Interactions between sex and plasma protein levels were assessed in the main pathophysiological model 2a. Because the MASP protein signal comprised peptides derived from different protein isoforms, we conducted sensitivity analyses on the association between the individual MASP peptides and incident type 2 diabetes. In another sensitivity analysis, we tested whether exclusion of the participants who converted directly from normoglycemia to incident type 2 diabetes affected the association estimates for incident prediabetes.

In the prediction analyses, we first selected the protein subsets which best predicted incident diabetes or incident (pre)diabetes according to the Akaike Information Criterion using logistic regression with stepwise variable selection on top of the following basic models: (1) GDRS_adapted_, (2) age + sex + HbA1c, (3) GDRS_adapted_ + HbA1c. For assessment of the predictive performance, we calculated the following metrics with 95% CIs using 10,000 bootstrap-samples as internal validation approach: (1) area under the receiver operating characteristic (ROC) curve (AUC) of the basic and protein-extended models and their difference (DeltaAUC), (2) integrated discrimination improvement (IDI) and (3) category-free net reclassification improvement (cfNRI) [28]. ROC-plots and risk assessment plots, which separately show the sensitivity of case prediction and the false positive rate of non-case-prediction over all possible risk cut-off values [29], were drawn.

Test results with two-sided *p* value < 0.05 were considered statistically significant.

## Results

### Descriptive statistics

Table 1 shows the characteristics of the study participants. The cases comprised more men than women and were on average older. At baseline, they were more likely to be physically inactive, to suffer from actual hypertension, and to have a parental and sibling history of diabetes. Furthermore, they had a higher waist circumference, a higher total cholesterol/HDL-cholesterol ratio, and higher triglyceride concentrations. At baseline and at follow-up cases had higher levels of fasting glucose, 2-h-glucose, fasting insulin, and HOMA-IR.

**Table 1:** Baseline characteristics of the study population

Characteristics	Incident type 2 diabetes			Incident (pre)diabetes		
	Non-cases^a^*n *= 660	Cases^b^*n *= 123	*p* value^e^	Non-cases^c^*n *= 446	Cases^d^*n *= 255	*p* value^e^
Male (%)	47.3	56.1	0.089	43.7	57.6	0.001
Characteristics at baseline						
Age (years)	57.4 ± 9.4	63.4 ± 8.6	<0.001	55.8 ± 9.1	59.9 ± 9.3	<0.001
Waist circumference (cm)	92.4 ± 13.8	102.2 ± 11.9	<0.001	90.0 ± 12.4	97.5 ± 11.0	<0.001
Height (cm)	169.0 ± 9.4	167.9 ± 9.2	0.377	169.0 ± 9.5	168.8 ± 9.5	0.976
Physically inactive (%)	35.5	51.2	0.001	33.6	41.6	0.044
Smoking (%)			0.035			0.880
Never	45.0	55.3		45.1	45.1	
Former	39.2	36.6		39.0	37.6	
Current	15.8	8.1		15.9	17.3	
Actual hypertension (%)	34.7	57.7	<0.001	26.9	47.1	<0.001
Parental history of diabetes (%)			0.010			<0.001
No	61.4	48.8		67.3	46.7	
Unknown	15.0	20.3		11.7	24.3	
One parent	21.4	24.4		19.5	25.1	
Both parents	2.3	6.5		1.6	3.9	
Sibling history of diabetes (%)	6.2	15.4	0.001	4.3	8.6	0.028
Triglyceride level (mmol/l)^f^	1.2 (0.8, 1.6)	1.6 (1.2, 2.2)	<0.001	1.1 (0.8, 1.5)	1.3 (0.9, 1.9)	<0.001
Total chol./HDL-cholesterol ratio^f^	3.9 (3.2, 4.6)	4.4 (3.8, 5.4)	<0.001	3.7 (3.1, 4.5)	4.2 (3.5, 4.9)	<0.001
HbA1c (%)	5.4 ± 0.3	5.8 ± 0.3	<0.001	5.3 ± 0.3	5.5 ± 0.3	<0.001
HbA1c (mmol/mol)	35.6 ± 3.4	39.8 ± 3.6	<0.001	34.9 ± 3.2	37.0 ± 3.2	<0.001
Fasting glucose^f,g^ (mmol/l)	5.2 (4.9, 5.5)	5.8 (5.4, 6.3)	<0.001	5.1 (4.8, 5.3)	5.4 (5.2, 5.7)	<0.001
2-h-glucose^f,g^ (mmol/l)	5.8 (4.9, 6.9)	8.3 (7.0, 9.2)	<0.001	5.4 (4.7, 6.3)	6.3 (5.5, 7.0)	<0.001
Fasting insulin^f,g^ (pmol/l)	49.8 (36.6, 66.0)	72.0 (53.4, 123.0)	<0.001	46.2 (34.2, 60.0)	60.0 (44.4, 78.0)	<0.001
HOMA-IR^f,g^	1.9 (1.4, 2.7)	3.1 (2.3, 5.2)	<0.001	1.7 (1.3, 2.3)	2.4 (1.8, 3.2)	<0.001
Characteristics at follow-up						
Fasting glucose^f,g^ (mmol/l)	5.4 (5.1, 5.8)	6.8 (6.0, 7.3)	<0.001	5.3 (5.0, 5.5)	6.0 (5.6, 6.3)	<0.001
2-h-glucose^f,g^ (mmol/l)	5.9 (5.0, 7.3)	11.8 (10.3, 13.0)	<0.001	5.5 (4.7, 6.2)	8.2 (7.3, 9.2)	<0.001
Fasting insulin^f,g^ (pmol/l)	55.4 (37.8, 81.0)	96.0 (66.0, 132.0)	<0.001	49.2 (35.3, 68.8)	74.9 (54.2, 104.5)	<0.001
HOMA-IR^f,g^	2.2 (1.5, 3.3)	4.8 (3.0, 6.9)	<0.001	1.9 (1.3, 2.8)	3.4 (2.3, 4.7)	<0.001

Percentages are given for categorical variables, arithmetic means ± SDs for approximately normally distributed, and median (25^th^; 75^th^ percentile) for skewed continuous variables

^a^Nondiabetic (fasting glucose < 7.0 mmol/l and 2-h-glucose ≤ 11.1 mmol/l) at baseline and follow-up

^b^Nondiabetic at baseline and known clinically diagnosed (*n *= 56) or newly OGTT diagnosed (*n *= 67) type 2 diabetes (fasting glucose ≥ 7.0 mmol/l and/or 2-h-glucose ≥ 11.1 mmol/l) at follow-up

^c^Normoglycemia (fasting glucose < 6.1 mmol/l and 2-h-glucose < 7.8 mmol/l) at baseline and follow-up

^d^Normoglycemia at baseline and prediabetes (*n *= 223) or known (*n *= 16) or newly diagnosed (*n *= 16) type 2 diabetes at follow-up (fasting glucose ≥ 6.1 mmol/l and/or 2-h-glucose ≥ 7.8 mmol/l)

^**e**^For differences between groups: Kruskal–Wallis test for continuous variables; χ^2^ test for categorical variables

^f^Skewed, continuous variables

^g^Descriptive statistics for the continuous type 2 diabetes related traits are only given for the study participants who were included in the linear regression analyses of these traits; number of non-cases/cases incident type 2 diabetes: *n *= 660/88 for fasting glucose, *n *= 660/64 for 2-h-glucose, *n *= 657/87 for fasting insulin and HOMA-IR; non-cases/cases (pre)diabetes: *n *= 446/247 for fasting glucose, *n *= 446/239 for 2-h-glucose, *n *= 445/246 for fasting insulin and HOMA-IR

### Analyses focused on pathophysiological mechanisms

**Fig. 2:**
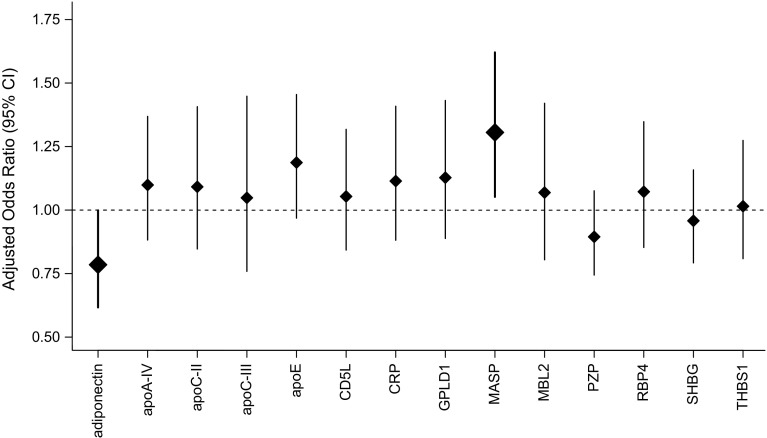
ORs with 95% CIs for incident type 2 diabetes per one sex-specific SD increase in SRM-MS measured proteins (*n *= 783), adjusted for age, sex, waist circumference, height, smoking, physical inactivity, actual hypertension, triglyceride level, and total cholesterol/HDL-cholesterol ratio (model 2a). Bars and diamonds of proteins associated statistically significantly with incident type 2 diabetes are printed in bold. *apoA-IV* apolipoprotein A-IV; *apoC-II* apolipoprotein C-II; *apoC-III* apolipoprotein C-III; *apoE* apolipoprotein E; *CD5L* CD5 molecule-like; *CRP* C-reactive protein; *GPLD1* glycosylphosphatidylinositol-specific phospholipase D1; *MASP* mannan-binding lectin serine peptidase; *MBL2* mannose-binding lectin 2; *PZP* pregnancy-zone protein; *RBP4* retinol-binding protein 4; *SHBG* sex hormone-binding globulin; *THBS1* thrombospondin 1

After adjustment for age, sex, waist and height (model 1), the levels of adiponectin were inversely and of apolipoprotein C-II (apoC-II), apoC-III, apoE, and MASP positively associated with incident type 2 diabetes (Supplemental Table 2). MASP levels were also positively associated with incident (pre)diabetes. After adjustment for additional risk factors (model 2a), adiponectin (OR per sex-specific SD: 0.785 [95% CI 0.617, 0.999] and MASP (1.306 [1.052, 1.621]) remained significantly associated with incident type 2 diabetes (Fig. 2); MASP also remained significantly associated with incident (pre)diabetes (1.241 [1.036, 1.486]). Adiponectin association estimates for incident diabetes and incident (pre)diabetes tended to be stronger in men than in women (*p* value_sex-interaction_ = 0.053 and 0.067, respectively), and the apoC-II diabetes association tended to be stronger in women (*p* value_sex-interaction_ = 0.077).

In the sensitivity analysis of the outcome incident prediabetes, in which we excluded the 32 study participants having progressed directly from normoglycemia to incident diabetes, the results remained essentially the same (Supplemental Table 3). In the sensitivity analysis investigating separate associations between the three measured MASP peptides and incident diabetes, the ‘TGVI’ peptide showed the strongest association (OR per sex-specific SD = 1.392 [95% CI 1.132, 1.711], model 2a), followed by ‘AAGN’ (1.260 [1.024, 1.551]) and ‘SLPT’ (1.104 [0.897, 1.359]).

**Fig. 3:**
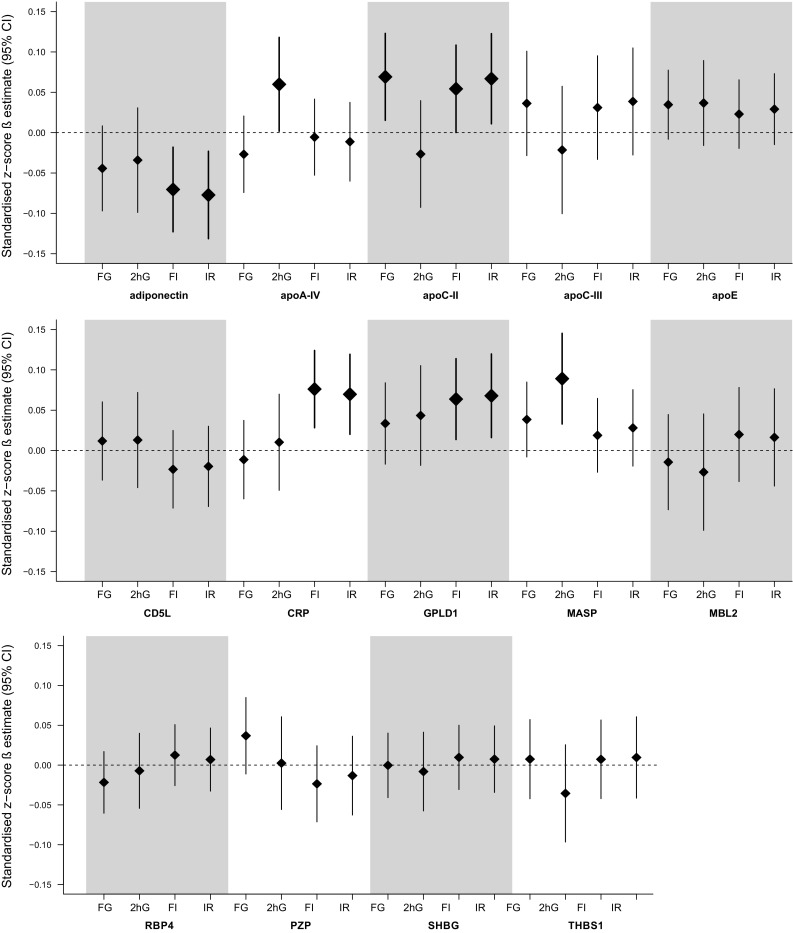
Estimated difference in continuous outcomes at follow-up for study participants not taking glucose-lowering medication expressed as the SD change in the continuous outcome (standardized *z*-score β estimate with 95% CI) per one sex-specific SD increase in the respective protein, adjusted for age, sex, waist circumference, height, smoking, physical inactivity, actual hypertension, triglyceride level, total cholesterol/HDL-cholesterol ratio (model 2a) and the baseline value of the investigated outcome variable. *FG* fasting glucose (*n *= 855); *2hG* 2-h-glucose (*n *= 831); *FI* fasting insulin (*n *= 851), *IR* HOMA-insulin resistance (*n *= 851). Bars and diamonds of proteins associated statistically significantly are printed in bold. *apoA-IV* apolipoprotein A-IV; *apoC-II* apolipoprotein C-II; *apoC-III* apolipoprotein C-III; *apoE* apolipoprotein E; *CD5L* CD5 molecule-like; *CRP* C-reactive protein; *GPLD1* glycosylphosphatidylinositol-specific phospholipase D1; *MASP* mannan-binding lectin serine peptidase; *MBL2* mannose-binding lectin 2; *PZP* pregnancy-zone protein; *RBP4* retinol-binding protein 4; *SHBG* sex hormone-binding globulin; *THBS1* thrombospondin 1

In the prospective analyses of the continuous outcomes, adiponectin levels were inversely associated with fasting insulin and HOMA-IR levels at follow-up (Fig. 3). Positive associations were observed for apoC-II with fasting glucose, fasting insulin and HOMA-IR, for C-reactive protein (CRP) as well as for glycosylphosphatidylinositol specific phospholipase D1 (GPLD1) with fasting insulin and HOMA-IR, and for apoA-IV and MASP with 2-h-glucose.

### Prediction analyses

**Fig. 4:**
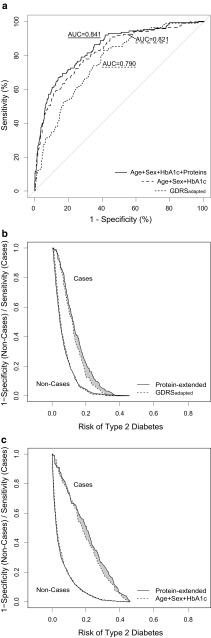
**a** Receiver operating characteristic (ROC) curves comparing main prediction models for incident type 2 diabetes. **b** Risk assessment plot for the GDRS_adapted_ prediction model, without (dashed lines) and with (solid lines) protein-extension. Lines in the lower left part of the figure represent 1-specificity for all possible risk cut-offs for non-cases; lines in the upper right part represent sensitivity for type 2 diabetes cases. The grey area represents the integrated discrimination improvement (IDI). **c** Risk assessment plot for the ‘Age + Sex + HbA1c’ prediction model, without (dashed lines) and with (solid lines) protein-extension. The non-case data was grossed up to represent the complete study cohort for the parts B and C of this figure in order to illustrate the relationship between risk of type 2 diabetes, sensitivity and specificity correctly. The ROC- and risk assessment plots were drawn using the complete study data without bootstrapping. Therefore, the AUC values displayed here deviate from the AUC_bootstrap_ values given in the text. All basic, extended and DeltaAUC values computed based on the complete study data are supplied in the Supplemental Table 4

The non-invasive GDRS-variables predicted incident type 2 diabetes with an AUC of 0.749 [0.687, 0.807] (Table 2). A subset of three out of all 14 proteins, namely MASP, adiponectin and apoE, was found to best improve the prediction on top of the non-invasive risk factors, with an AUC of 0.772 [0.712, 0.828]. Figure 4a illustrates the AUC values estimated using the complete study data without bootstrapping because they cannot be adequately drawn for the bootstrap-approach.

**Table 2:** Prediction performance of selected proteins for incident type 2 diabetes, calculated using 10,000 bootstrap-samples, *n *= 783

Basic prediction model	Selected proteins	Basic AUC (95% CI)	Extended AUC (95% CI)	Delta AUC (95% CI)	IDI overall (95% CI)	IDI cases (95% CI)	IDI controls (95% CI)	cfNRI overall (95% CI)	cfNRI cases (95% CI)	cfNRI controls (95% CI)
GDRS_adapted_^a^	MASP, adiponectin, apoE	0.749 (0.687, 0.807)	0.772 (0.712, 0.828)	0.023 (–0.014, 0.051)	**0**.**031** (**0**.**004**, **0**.**059**)	0.026 (–0.004, 0.058)	0.005 (–0.006, 0.015)	**0**.**393** (**0**.**103**, **0**.**680**)	0.119 (–0.179, 0.409)	**0**.**274** (**0**.**114**, **0**.**427**)
Age + sex + HbA1c	MASP, adiponectin, apoE	0.816 (0.759, 0.870)	0.828 (0.773, 0.881)	0.012 (–0.014, 0.031)	**0**.**030** (**0**.**000**, **0**.**059**)	0.025 (–0.007, 0.059)	0.004 (–0.006, 0.014)	**0**.**400** (**0**.**116**, **0**.**682**)	0.126 (–0.163, 0.415)	**0**.**275** (**0**.**108**, **0**.**434**)
GDRS_adapted_ + HbA1c^b^	MASP, adiponectin, apoE, PZP	0.823 (0.768, 0.875)	0.828 (0.775 0.879)	0.005 (–0.019, 0.024)	0.025 (–0.006, 0.057)	0.021 (–0.012, 0.057)	0.003 (–0.006, 0.013)	**0**.**358** (**0**.**050**, **0**.**651**)	0.067 (–0.231, 0.364)	**0**.**291** (**0**.**116**, **0**.**460**)

Statistically significant results are printed in bold

*AUC* area under the receiver operating characteristic curve: basic AUC without proteins, extended AUC with selected proteins, *IDI* integrated discrimination improvement of protein-extended versus basic model, *cfNRI* category-free net reclassification improvement of protein-extended versus basic model

^a^Model 3a: GDRS_adapted_ prediction variables: age, sex, waist circumference, height, smoking, physical inactivity, actual hypertension, parental history of diabetes, sibling history of diabetes

^b^Model 3b: Model 3a variables plus HbA1c concentrations

The same protein panel also best improved diabetes prediction compared to our second reference model consisting of age, sex, and HbA1c. The AUC was 0.816 [0.759, 0.870] for the basic and 0.828 [0.773, 0.881] for the protein-extended model. Compared to the protein-extended HbA1c-model, the model which additionally included the GDRS_adapted_-variables did not yield a higher AUC (0.828 [0.775, 0.879]).

The IDI and the cfNRI metrics confirmed the predictive value of the protein panel for type 2 diabetes on top of the GDRS-variables (IDI = 0.031 [95% CI 0.004, 0.059]; cfNRI = 0.393 [95% CI 0.103, 0.680]) and also on top of age, sex, and HbA1c (IDI = 0.030 [0.000, 0.059]; cfNRI = 0.400 [0.116, 0.682]) (Table 2). While the cfNRI describes the proportion of individuals for whom the change in calculated risk was in the desired direction (higher for cases, lower for non-cases), the IDI quantifies the actual change in calculated risk for each individual (sensitivity for cases and 1-specificity for non-cases). For both reference models the gain in prediction performance mainly consisted of an improved sensitivity to identify diabetes cases (Fig. 4b, c).

The predictive performance for incident (pre)diabetes of the GDRS_adapted_-variables (AUC = 0.723 [0.671, 0.773]) and of age, sex, and HbA1c (0.719 [0.669, 0.771]) was lower than for diabetes (Table 3). For the GDRS_adapted_ reference model only MASP and for the age, sex, and HbA1c reference model, the combination of MASP and CRP was selected as predictor in stepwise logistic regression analysis. While MASP did not add statistically significant information on top of the GDRS_adapted_-variables, MASP combined with CRP improved the (pre)diabetes prediction on top of age, sex, and HbA1c with an IDI of 0.021 [0.005, 0.036] and a cfNRI of 0.270 [0.051, 0.481].

**Table 3:** Prediction performance of selected proteins for incident (pre)diabetes, calculated using 10,000 bootstrap-samples, *n *= 701

Basic prediction model	Selected proteins	Basic AUC (95% CI)	Extended AUC (95% CI)	Delta AUC (95% CI)	IDI overall (95% CI)	IDI cases (95% CI)	IDI controls (95% CI)	cfNRI overall (95% CI)	cfNRI cases (95% CI)	cfNRI controls (95% CI)
GDRS_adapted_^a^	MASP	0.723 (0.671, 0.773)	0.725 (0.674, 0.775)	0.002 (–0.017, 0.012)	0.006 (–0.004, 0.014)	0.004 (–0.007, 0.014)	0.002 (–0.006, 0.009)	0.097 (–0.145, 0.322)	0.032 (–0.191, 0.250)	0.065 (–0.097, 0.227)
Age + sex + HbA1c	MASP, CRP	0.719 (0.669, 0.771)	0.731 (0.681, 0.781)	0.012 (− 0.022, 0.033)	**0**.**021** (**0**.**005**, **0**.**036**)	0.013 (− 0.004, 0.031)	0.008 (− 0.006, 0.020)	**0**.**270** (**0**.**051**, **0**.**481**)	0.100 (− 0.111, 0.308)	**0**.**170** (**0**.**012**, **0**.**326**)
GDRS_adapted_ + HbA1c^b^	MASP	0.754 (0.704, 0.802)	0.757 (0.707, 0.805)	0.003 (–0.014, 0.013)	0.006 (–0.004, 0.016)	0.004 (–0.008, 0.015)	0.002 (–0.006, 0.010)	0.122 (–0.116, 0.347)	0.046 (–0.176, 0.263)	0.076 (–0.090, 0.239)

Statistically significant results are printed in bold

*AUC* area under the receiver operating characteristic curve: basic AUC without proteins, extended AUC with selected protein (MASP), *IDI* integrated discrimination improvement of protein-extended versus basic model, *cfNRI* category-free net reclassification improvement of protein-extended versus basic model

^a^Model 3a: GDRS_adapted_ prediction variables: age, sex, waist circumference, height, smoking, physical inactivity, actual hypertension, parental history of diabetes, sibling history of diabetes

^b^Model 3b: Model 3a variables plus HbA1c concentrations

## Discussion

This large SRM-MS-based cohort study discovered a novel association between MASP protein levels and both incident type 2 diabetes as well as incident (pre)diabetes even after adjustment for established risk factors and biomarkers. This means that MASP levels are not only elevated relatively shortly before the onset of type 2 diabetes but already in those normoglycemic individuals who will progress to (pre)diabetes during the next 6.5 years. The results of this study also clearly show that MASP together with apoE and adiponectin improves the prediction of type 2 diabetes on top of non-invasive risk factor variables and on top of age, sex, and HbA1c concentrations, which are well-known to have a high predictive power [30].

### MASP signal

Our previous cross-sectional investigation of the KORA F4 study showed that, compared to normoglycemic persons, prediabetic and diabetic cases had higher MASP plasma levels. Moreover, higher MASP levels were associated with both higher fasting and 2-h-glucose levels [23]. Krogh and coworkers have recently confirmed this cross-sectional association by reporting higher MASP-1 levels in persons with type 2 diabetes compared to controls [31].

The current prospective investigation, in which we newly measured protein data for more than two thirds of the studied persons, adds that MASP plasma levels are already elevated years before type 2 diabetes or (pre)diabetes developed. The potential mechanistic link is currently unclear, but non-traditional roles of complement proteins, such as involvement in type 2 diabetes associated inflammation, beta-cell secretory function and maintaining homeostasis of the pancreatic islets have been suggested [32]. In our data MASP was specifically associated with elevated 2-h-glucose concentrations suggesting that particularly insulin secretion after glucose stimulation may be impaired.

Since our MASP signal stems from peptides which are proteotypic for the three isoforms MASP-1, MASP-3, and MAP44, a clear discrimination between these isoforms is currently not possible. MASP-1 is the most abundant serine protease of the complement lectin pathway and thus a major player in the complement cascade which is initiated when a complex comprising mannose-binding lectin (MBL), MBL-associated serine proteases (MASPs: MASP-1, MASP-2, MASP-3) and MBL-associated proteins (MAP19 and MAP44) binds to its target carbohydrate-containing ligands, primarily derived from pathogens or damaged tissues [33, 34]. In our peptide-specific sensitivity analysis, the ‘TGVI’ and ‘AAGN’ peptides were most strongly associated with incident diabetes. In contrast to the ‘SLPT’ peptide which is proteotypic solely for the MASP-1 isoform, these two peptides can derive from MASP-1, MASP-3 or MAP44, suggesting that not MASP-1 but rather MASP-3 or MAP44 are responsible for the observed association and should also be most useful for prediction purposes. As compared to MASP-1 and MASP-2, MASP-3 has a distinct substrate specificity and inhibitor profile and for instance selectively cleaves insulin-like growth factor (IGF) binding protein 5 (IGFBP-5) which binds to IGFs such as IGF-1. MASP-3 may thus modulate the interaction between IGFs and their receptors on cell surfaces [35]. Circulating IGF-1 has been shown to correlate negatively with HOMA-IR in the Framingham Heart Study [36].

### Further mechanistic implications

While it has been known for a long time that triglycerides, total cholesterol and HDL cholesterol are associated with type 2 diabetes risk, the association between different apolipoprotein components and type 2 diabetes has only recently been addressed. In the present study apoE, apoC-II and apoC-III levels were higher in those study participants who later developed incident type 2 diabetes, when adjusted for age, sex, and anthropometric measures. This confirms previous studies for apoE [37] and apoC-III [37, 38]. All three apolipoproteins correlate positively with the total cholesterol/HDL-cholesterol ratio and triglycerides [23] (although this fact is not completely understood for apoC-II, because this apolipoprotein is an essential cofactor for the lipoprotein lipase which mediates triglyceride hydrolysis [39]). In order to assess whether the apolipoproteins are associated with incident diabetes independently of these known risk factors, we adjusted for these lipids as a next step. The adjustment attenuated all associations which corresponds to previous results observed for apoE but not for apoC-III [37]; the reason might be that in contrast to the previous study, we adjusted not only for triglycerides but also for the total cholesterol/HDL-cholesterol ratio.

Interestingly, apoC-II, which to our knowledge has not been investigated for association with incident type 2 diabetes to date, showed the strongest positive associations with fasting glucose, fasting insulin and HOMA-IR of all investigated apolipoproteins in the present study. Several pathways have been suggested to link apolipoproteins with diabetes risk. Apart from the pathogenic role of higher triglyceride levels associated with higher apolipoprotein levels [39], and their possible role in inflammatory pathways [38], it was shown in animal and cell studies that apoC-III may promote the development of diabetes directly, by interfering with both function and survival of pancreatic beta-cells [40].

The inverse association between concentrations of the adipose-tissue derived hormone adiponectin and incident type 2 diabetes observed in the present study is well established and confirms our own previous investigations using a different measurement technology [4] and work from other groups [41].

### Predictive value of proteins

Regarding our prediction results, several issues deserve to be highlighted. First, the HbA1c reference model predicted type 2 diabetes substantially better than the GDRS_adapted_-variables alone. Nevertheless, MASP, apoE, and adiponectin improved the prediction on top of HbA1c, age and sex to a similar extent in terms of the IDI- and NRI-metrics as on top of the GDRS_adapted_ reference model. Second, the combination of the circulating biomarkers MASP, apoE, adiponectin, HbA1c, age and sex predicted diabetes equally well as the GDRS_adapted_-variables and HbA1c. We therefore conclude that while the non-invasive GDRS was mainly developed for self-assessment of one’s own risk of type 2 diabetes development [42], biomarker information may strongly improve the predictive power. HbA1c combined with information on age and sex is highly predictive, and the additional assessment of the circulating proteins MASP, apoE and adiponectin may improve the prediction even further.

Other recent unbiased biomarker prediction studies using different measurement platforms have also identified promising candidates for type 2 diabetes prediction such as ferritin, α-hydroxybutyrate, or α-tocopherol [9, 10]. Combining these markers with the novel markers identified in the present study may further improve the prediction. However, this needs to be investigated in additional studies as the comparison of the predictive performance across different published studies is hampered by use of different methodology, especially by use of different study populations, reference models and prediction measures [43]. Moreover, the clinical relevance of the biomarkers’ gain in predictive power will depend on the availability of cost-efficient measurement devices.

Concerning (pre)diabetes, both reference models had similar predictive power in terms of AUCs, but overall the AUCs for the prediction of incident (pre)diabetes were substantially lower as compared to incident diabetes. The proteins’ predictive power was also lower. On top of the non-invasive GDRS_adapted_-variables, there was no evidence of improved prediction. However, on top of HbA1c, age, and sex, MASP combined with CRP improved (pre)diabetes prediction significantly in terms of both IDI- and cfNRI-metrics. We are aware of only one other study that has assessed the utility of biomarkers to predict incident prediabetes [12]. This study investigated electronic health record data and also found that CRP levels (besides HDL cholesterol and alanine aminotransferase) predicted the progression from normoglycemia to prediabetes, though no information on statistical significance of the prediction improvement was reported.

### Strengths and limitations

To our knowledge, this study is the largest SRM-MS-based proteomics biomarker study in the field of type 2 diabetes research. An additional strength is the prospective design with OGTT data available at baseline and follow-up. This enabled us to investigate (1) who progressed to type 2 diabetes (including newly diagnosed diabetes) among all participants who were nondiabetic at baseline, (2) who converted to (pre)diabetes among all participants who were normoglycemic at baseline, and (3) prospective associations with continuous glucose and insulin outcomes. The availability of many established type 2 diabetes risk factors and HbA1c concentrations made adjustment of our association analyses for the most relevant confounders and selection of appropriate reference models for prediction benchmarking possible. Finally, we assessed the predictive power of the proteins thoroughly, using several metrics and plots as recommended [43].

A limitation is that our approach did not provide absolute protein concentrations, which, however, should not affect the reported associations. Moreover, it was unclear to which protein isoform the MASP signal belonged. Nevertheless, the signal can be used for prediction purposes, but future studies should clarify which protein isoform or combination thereof is physiologically most relevant. Another limitation is the use of an adapted version of the GDRS as reference model, which most probably lead to a slightly lower basic predictive performance compared to the originally proposed model. In our second reference model, we used HbA1c together with age and sex information amongst others because HbA1c concentrations are not affected by food intake. However, because our study only comprised fasting blood sampling, we could not investigate whether the predictive power of our selected proteins would be equally high using non-fasting samples. Finally, although this study used a state-of-the-art statistical technique for internal validation of the prediction performance, no replication in an independent study has been conducted. Therefore, and because we did not adjust our analyses for multiple testing due to our relatively small sample size (compared to other epidemiological studies investigating single or only few markers), corroboration of our findings will be necessary.

## Conclusion

In summary, we report a novel association of increased MASP plasma protein levels with incident type 2 diabetes and incident prediabetes, independent of established type 2 diabetes risk factors. In combination with apoE and adiponectin, MASP improved the prediction of type 2 diabetes beyond non-invasive risk factor variables and beyond HbA1c, age, and sex. External replication and cost-effectiveness studies will need to assess the clinical relevance of the proteins’ gain in predictive power.

## Electronic supplementary material

Below is the link to the electronic supplementary material.

Supplementary material 1 (PDF 2,630 kb)
