# Supplementary material for: Protein markers and risk of type 2 diabetes and prediabetes: a targeted proteomics approach in the KORA F4/FF4 study
Source: Eur J Epidemiol. 2018 Dec 31;34(4):409–22. doi: 10.1007/s10654-018-0475-8 (PMC6451724; doi:10.1007/s10654-018-0475-8)
Supplement: Supplementary file 1 — Supplementary material 1 (PDF 2,630 kb) [file 10654_2018_475_MOESM1_ESM.pdf]

Online-Only Supplemental Material

related to the Article

Protein markers and risk of type 2 diabetes and prediabetes: a targeted proteomics approach in the KORA F4/FF4 study

Authors: Cornelia Huth[1,2], Christine von Toerne, Florian Schederecker, Tonia de las Heras Gala, Christian Herder, Florian Kronenberg, Christa Meisinger, Wolfgang Rathmann, Wolfgang Koenig, Melanie Waldenberger, Michael Roden, Annette Peters, Stefanie M. Hauck, Barbara Thorand

[1]Institute of Epidemiology, Helmholtz Zentrum München – German Research Center for Environmental Health (GmbH), Neuherberg, Germany

[2]German Center for Diabetes Research (DZD), München-Neuherberg, Germany

Corresponding author:

Cornelia Huth, PhD, MSE, Dipl.oec.troph.

e-mail: huth@helmholtz-muenchen.de

Targeted SRM-MS protein measurements *(the description here in the Supplemental Material is complete and contains – in contrast to the description in the main article – some technical details which are not necessary to understand the article; the description here is intentionally partly redundant to the description in the main article)*

The plasma samples of 271 persons of the subcohort had already been measured previously (lot 1) [1]. The remaining subcohort and all additional incident cases were newly measured in 2016 when the FF4 data became available (lot 2, *n*=621). These measurements were performed similarly as published for lot 1 [1] and are described together with the combined data preprocessing in detail below.

Plasma depletion and on-bead digestions The 621 lot 2 samples were randomly distributed into thirteen processing batches and quality control was performed as described for lot 1 [1]. All lot 2 sample preparation batches passed quality control. Depletion using ProteoMiner beads and tryptic digestion of lot 2 plasma were performed as previously described [1, 2] in the thirteen batches. After digestion, samples were stored at −80°C.

MS measurement and relative quantification Selected peptides established in [1] were measured by mass spectrometry (MS) in a targeted selection reaction monitoring (SRM) approach. SRM-assays were created for 24 proteins, including one spike-in control protein as

described in [1]. Isotope-labelled synthetic (heavy) peptides were used for each peptide as internal control to correct signal integration and for relative quantification as described in [1]. The heavy peptide mix was added to the digested sample prior to MS measurement. Liquid chromatography MSMS analysis was performed on an Ultimate3000 HPLC system (Thermo Fischer Scientific, Dreieich, Germany) coupled online to a QTrap4000 (ABSCIEX, Framingham, MT, USA) mass spectrometer by a nanospray ion source, as previously described [1] with the following changes: Peptides were separated on a 60 min non-linear gradient of 5% acetonitrile in 0.1% formic acid to 40% at a flow rate of 300 nl/min and the interface heater temperature was set to 150 °C. The signals were quantified by calculation of the area under the curve (AUC) of pre-specified collision-induced peptide dissociation products, the so-called transitions using the software Skyline. All transitions were monitored (and manually corrected if necessary) for correct peak integration and for identical retention time of associated light and heavy signals.

Relative quantification, normalization and quality control Data preprocessing was performed using R version 3.4.2 [3]. Coefficients of variation (CV) of pooled samples were calculated for all transition signals based on five replicate measurements per lot using the software AuDIT [4]. Transitions with a CV ≥30% were excluded. A few measurements that were zero were randomly assigned a number between 1 and the minimum observation of the respective transition. Reduction from three to two transitions per peptide was performed as described [1]. Light (endogenous) to heavy (synthetic) ratios (LHRs) of the AUC-values were calculated, $\log_2$-transformed, and averaged for all transitions of each peptide. Peptide-level LHR information was averaged per protein to yield relative protein levels. Within this process, the data was corrected for the technical covariates lot and batch, instrument maintenance and cleaning routines, day of analysis, time on autosampler and spike-in control peptide measurement results. Quality control of signals was based on CV results of lot 1 and lot 2 pools and 29 duplicate measurements of lot 2 samples. Only peptides demonstrating reliable reproducibility were included in the combined analysis, leaving a total of 30 peptides in lot 1 and 31 peptides in lot 2, and representing 14 candidate proteins (Supplemental Table 1). The SRM-MS signals of all analyzed transitions are shown in Supplemental Fig. 2 exemplarily for a plasma sample pool. The work-flow from plasma depletion, via SRM-MS measurement, to computation of multivariable adjusted odds ratios (ORs) is illustrated in Supplemental Fig. 3.

GDRS adaptations

In contrast to the originally published version [5, 6], the adapted version of the GDRS used in the present analysis did not include dietary information on meat, fiber and coffee consumption, because this data was unavailable in KORA F4. Additionally, smoking status was analyzed using three instead of the originally proposed five categories to avoid statistically instable results due to small number of participants in some categories, and the physical inactivity variable only included sports but not gardening and biking. Regarding parental history, we included the additional category 'unknown', which incorporates a higher type 2 diabetes risk, separately from the category 'no parental history' [7]. Although the latter more detailed assessment should individually result in a higher predictive performance, altogether the aforementioned necessary adaptations are likely to lead to a slightly lower predictive performance of the used $GDRS_{adapted}$ reference model compared to the originally proposed model.

Supplemental References

1. von Toerne C, Huth C, de Las Heras Gala T, Kronenberg F, Herder C, Koenig W et al. MASP1, THBS1, GPLD1 and ApoA-IV are novel biomarkers associated with prediabetes: the KORA F4 study. Diabetologia. 2016;59:1882-92.

2. von Toerne C, Kahle M, Schafer A, Ispiryan R, Blindert M, Hrabe De Angelis M et al. Apoe, Mbl2, and Psp Plasma Protein Levels Correlate with Diabetic Phenotype in NZO Mice - An Optimized Rapid Workflow for SRM-Based Quantification. J Proteome Res. 2013;12:1331-43.

3. R Core Team. R: A Language and Environment for Statistical Computing. R Foundation for Statistical Computing; 2017.

4. Abbatiello SE, Mani DR, Keshishian H, Carr SA. Automated detection of inaccurate and imprecise transitions in peptide quantification by multiple reaction monitoring mass spectrometry. Clin Chem. 2010;56:291-305.

5. DIfE - Deutscher Diabetes-Risiko-Test (DRT). Diabetologie und Stoffwechsel. 2016;11:S204-S6.

6. Paprott R, Muhlenbruch K, Mensink GB, Thiele S, Schulze MB, Scheidt-Nave C et al. Validation of the German Diabetes Risk Score among the general adult population: findings from the German Health Interview and Examination Surveys. BMJ Open Diabetes Res Care. 2016;4:e000280.

7. Thorand B, Liese AD, Metzger MH, Reitmeir P, Schneider A, Lowel H. Can inaccuracy of reported parental history of diabetes explain the maternal transmission hypothesis for diabetes? Int J Epidemiol. 2001;30:1084-9.

SUPPLEMENTAL TABLES

Supplemental Table 1 Plasma proteins and their peptides, measured by SRM-MS and investigated for association with incident type 2 diabetes and incident prediabetes

**Protein ID and Peptide sequence** [amino acid position]	**Protein Symbol**	**Protein Name**
**ENSP00000320709**	ADIPOQ	adiponectin
K.GDIGETGVPGAEGPR.G [77, 91]		
**ENSP00000350425**	apoA-IV	apolipoprotein A-IV
K.SELTQQLNALFQDK.L [51, 64]		
K.LGEVNTYAGDLQK.K [65, 77]		
R.ISASAEELR.Q [255, 263]		
**ENSP00000466775**	apoC-II	apolipoprotein C-II
K.ESLSSYWESAK.T [41, 51]		
K.TAAQNLYEK.T [52, 60]		
K.TYLPAVDEK.L [61, 69]		
**ENSP00000227667**	apoC-III	apolipoprotein C-III
R.GWVTDGFSSLK.D [60, 70]		
**ENSP00000252486**	apoE	apolipoprotein E
K.SELEEQLTPVAEETR.A [93, 107]		
R.AATVGSLAGQPLQER.A [209, 223]		
K.LEEQAQQIR.L [260, 268]		
**ENSP00000357156**	CD5L	CD5 molecule-like
R.EATLQDCPSGPWGK.N [211, 224]		
K.NTCNHDEDTWVECEDPFDLR.L [225, 244]		
R.LVGGDNLCSGR.L [245, 255]		
**ENSP00000255030**	CRP	C-reactive protein, pentraxin-related
K.ESDTSYVSLK.A [31, 40]		
R.GYSIFSYATK.R [65, 74]		
**ENSP00000230036**	GPLD1	glycosylphosphatidylinositol specific phospholipase D1
R.IADVTSGLIGGEDGR.V [742, 756][a]		
K.AQYVLISPEASSR.F [786, 798]		
R.FGSSLITVR.S [799, 807][b]		
**ENSP00000336792/ENSP00000296280**	MASP	mannan-binding lectin serine peptidase
R.TGVITSPDFPNPYPK.S [193, 207]		
R.AAGNECPELQPPVHGK.I [295, 310]		
R.SLPTCLPVCGLPK.F [427, 439]		
**ENSP00000363079**	MBL2	mannose-binding lectin (protein C) 2, soluble
K.WLTFSLGK.Q [124, 131][b]		
K.FQASVATPR.N [157, 165][b]		
R.NAAENGAIQNLIK.E [166, 178]		

Supplemental Table 1 – *continued*

Protein ID and Peptide sequence [amino acid position]	Protein Symbol	Protein Name
**ENSP00000261336**	PZP	pregnancy-zone protein
K.GSFALSFPVESDVAPIAR.M [517, 534]		
K.TLLVEAEGIEQEK.T [907, 919]		
**ENSP00000360519**	RBP4	retinol binding protein 4, plasma
K.YWGVASFLQK.G [107, 116][a]		
R.LIVHNGYCDGR.S [184, 194]		
**ENSP00000369816**	SHBG	sex hormone-binding globulin
R.IALGGLLFPASNLR.L [169, 182]		
K.QAEISASAPTSLR.S [202, 214]		
**ENSP00000260356**	THBS1	thrombospondin 1
K.GGVNDNFQGVLQNVR.F [201, 215]		
R.TIVTTLQDSIR.K [288, 298]		
**Control protein**	Rubisco	ribulose-1,5-bisphosphate carboxylase oxygenase (spinach)
K.TFQGPPHGIQVER.D [146, 158]		
R.FLFCAEALYK.A [217, 226]		
R.DITLGFVDLLR.D [339, 349]		
R.VALEACVQAR.N [421, 430]		

[a]Exclusively used in lot 1 data

[b]Exclusively used in lot 2 data

Supplemental Table 2 Multivariable adjusted logistic regression results for association between the 14 proteins (per one sex-specific SD increase) and incident type 2 diabetes as well as incident prediabetes.

Protein<br>Adjustment	Incident Type 2 Diabetes vs Non-Cases (n=123 vs 660)			Incident (Pre)diabetes[a] vs Non-Cases (n=255 vs 446)		
	OR	95% CI	*p* value	OR	95% CI	*p* value
adiponectin						
Model 1	**0.713**	**[0.570, 0.893]**	**0.003**	0.936	[0.778, 1.127]	0.486
Model 2a	**0.785**	**[0.617, 0.999]**	**0.049**	0.994	[0.820, 1.205]	0.951
Model 2b	0.843	[0.652, 1.090]	0.193	1.043	[0.854, 1.273]	0.682
Model 3a	**0.709**	**[0.561, 0.894]**	**0.004**	0.996	[0.824, 1.205]	0.970
Model 3b	**0.773**	**[0.605, 0.986]**	**0.038**	1.052	[0.863, 1.282]	0.614
apoA-IV						
Model 1	1.056	[0.851, 1.311]	0.621	1.064	[0.892, 1.268]	0.490
Model 2a	1.099	[0.882, 1.369]	0.400	1.064	[0.890, 1.270]	0.497
Model 2b	1.016	[0.802, 1.288]	0.892	0.986	[0.821, 1.185]	0.885
Model 3a	1.077	[0.861, 1.347]	0.514	1.043	[0.870, 1.250]	0.647
Model 3b	0.994	[0.782, 1.263]	0.960	0.973	[0.808, 1.172]	0.773
apoC-II						
Model 1	**1.279**	**[1.029, 1.591]**	**0.027**	1.064	[0.895, 1.264]	0.484
Model 2a	1.092	[0.847, 1.407]	0.499	1.037	[0.843, 1.274]	0.733
Model 2b	1.113	[0.845, 1.466]	0.447	1.021	[0.825, 1.262]	0.851
Model 3a	**1.309**	**[1.046, 1.637]**	**0.018**	1.037	[0.867, 1.240]	0.688
Model 3b	**1.288**	**[1.009, 1.643]**	**0.042**	1.009	[0.838, 1.214]	0.928
apoC-III						
Model 1	**1.302**	**[1.009, 1.681]**	**0.042**	1.041	[0.854, 1.269]	0.691
Model 2a	1.048	[0.759, 1.448]	0.775	1.002	[0.781, 1.284]	0.990
Model 2b	1.110	[0.784, 1.571]	0.556	0.976	[0.755, 1.263]	0.855
Model 3a	**1.376**	**[1.056, 1.794]**	**0.018**	1.028	[0.838, 1.262]	0.791
Model 3b	**1.364**	**[1.026, 1.812]**	**0.032**	0.998	[0.805, 1.236]	0.983
apoE						
Model 1	**1.318**	**[1.093, 1.589]**	**0.004**	1.131	[0.974, 1.315]	0.107
Model 2a	1.187	[0.968, 1.455]	0.099	1.107	[0.944, 1.298]	0.213
Model 2b	**1.267**	**[1.013, 1.583]**	**0.038**	1.126	[0.956, 1.326]	0.156
Model 3a	**1.329**	**[1.095, 1.612]**	**0.004**	1.094	[0.936, 1.279]	0.257
Model 3b	**1.376**	**[1.112, 1.702]**	**0.003**	1.103	[0.938, 1.297]	0.235
CD5L						
Model 1	1.070	[0.862, 1.329]	0.539	1.048	[0.882, 1.245]	0.592
Model 2a	1.054	[0.842, 1.318]	0.647	1.084	[0.908, 1.294]	0.371
Model 2b	1.171	[0.917, 1.497]	0.206	1.133	[0.943, 1.361]	0.183
Model 3a	1.066	[0.854, 1.330]	0.573	1.089	[0.910, 1.303]	0.352
Model 3b	1.171	[0.918, 1.494]	0.203	1.132	[0.940, 1.363]	0.192

Supplemental Table 2 – *continued*

Protein<br>Adjustment	Incident Type 2 Diabetes vs Non-Cases (*n*=123 vs 660)			Incident (Pre)diabetes[a] vs Non-Cases (*n*=255 vs 446)		
	OR	95% CI	*p* value	OR	95% CI	*p* value
CRP						
Model 1	1.142	[0.913, 1.428]	0.244	1.117	[0.934, 1.336]	0.224
Model 2a	1.114	[0.881, 1.409]	0.366	1.069	[0.889, 1.285]	0.479
Model 2b	1.040	[0.808, 1.339]	0.760	1.097	[0.907, 1.327]	0.342
Model 3a	1.161	[0.919, 1.465]	0.210	1.105	[0.916, 1.333]	0.296
Model 3b	1.067	[0.830, 1.372]	0.612	1.129	[0.930, 1.369]	0.219
GPLD1						
Model 1	1.156	[0.917, 1.457]	0.221	1.114	[0.927, 1.339]	0.251
Model 2a	1.128	[0.888, 1.432]	0.324	1.126	[0.930, 1.362]	0.223
Model 2b	1.164	[0.910, 1.489]	0.228	1.116	[0.920, 1.355]	0.266
Model 3a	1.182	[0.932, 1.499]	0.169	1.127	[0.930, 1.366]	0.222
Model 3b	1.206	[0.942, 1.542]	0.137	1.111	[0.914, 1.352]	0.290
MASP						
Model 1	**1.358**	**[1.100, 1.677]**	**0.004**	**1.232**	**[1.033, 1.470]**	**0.020**
Model 2a	**1.306**	**[1.052, 1.621]**	**0.016**	**1.241**	**[1.036, 1.486]**	**0.019**
Model 2b	**1.274**	**[1.013, 1.601]**	**0.038**	**1.284**	**[1.063, 1.551]**	**0.009**
Model 3a	**1.375**	**[1.112, 1.702]**	**0.003**	**1.239**	**[1.031, 1.490]**	**0.022**
Model 3b	**1.341**	**[1.068, 1.683]**	**0.011**	**1.277**	**[1.053, 1.549]**	**0.013**
MBL2						
Model 1	1.075	[0.814, 1.421]	0.609	0.997	[0.804, 1.237]	0.981
Model 2a	1.069	[0.804, 1.420]	0.646	0.969	[0.780, 1.205]	0.778
Model 2b	1.122	[0.818, 1.539]	0.475	0.967	[0.769, 1.214]	0.770
Model 3a	1.052	[0.793, 1.396]	0.727	0.964	[0.771, 1.205]	0.747
Model 3b	1.094	[0.802, 1.493]	0.569	0.965	[0.763, 1.220]	0.765
PZP						
Model 1	0.879	[0.734, 1.053]	0.161	1.049	[0.906, 1.213]	0.522
Model 2a	0.895	[0.744, 1.076]	0.237	1.038	[0.895, 1.204]	0.622
Model 2b	0.863	[0.706, 1.054]	0.149	1.040	[0.892, 1.213]	0.613
Model 3a	0.878	[0.731, 1.056]	0.167	1.062	[0.914, 1.235]	0.429
Model 3b	0.841	[0.689, 1.027]	0.089	1.064	[0.910, 1.245]	0.436
RBP4						
Model 1	1.173	[0.944, 1.457]	0.150	0.933	[0.787, 1.106]	0.427
Model 2a	1.072	[0.853, 1.348]	0.551	0.939	[0.785, 1.123]	0.489
Model 2b	1.032	[0.802, 1.327]	0.809	0.958	[0.796, 1.152]	0.648
Model 3a	1.159	[0.930, 1.443]	0.188	0.969	[0.813, 1.155]	0.729
Model 3b	1.124	[0.882, 1.433]	0.344	0.983	[0.820, 1.178]	0.850
SHBG						
Model 1	0.863	[0.723, 1.031]	0.105	0.988	[0.852, 1.144]	0.867
Model 2a	0.958	[0.792, 1.158]	0.656	0.991	[0.851, 1.155]	0.911
Model 2b	1.146	[0.928, 1.416]	0.204	1.040	[0.889, 1.216]	0.624
Model 3a	0.875	[0.728, 1.052]	0.155	0.943	[0.808, 1.099]	0.450
Model 3b	1.043	[0.853, 1.276]	0.681	0.991	[0.846, 1.161]	0.910

Supplemental Table 2 *continued*

Protein<br>Adjustment	Incident Type 2 Diabetes vs Non-Cases (*n*=123 vs 660)			Incident (Pre)diabetes[a] vs Non-Cases (*n*=255 vs 446)		
	OR	95% CI	*p* value	OR	95% CI	*p* value
THBS1						
Model 1	1.010	[0.810, 1.259]	0.931	1.039	[0.865, 1.247]	0.685
Model 2a	1.015	[0.809, 1.274]	0.897	1.016	[0.843, 1.223]	0.870
Model 2b	0.966	[0.760, 1.226]	0.774	0.997	[0.823, 1.208]	0.977
Model 3a	1.035	[0.825, 1.299]	0.765	1.011	[0.836, 1.223]	0.912
Model 3b	0.980	[0.772, 1.245]	0.870	0.991	[0.815, 1.205]	0.924

Statistically significant results are printed in bold.

Model 1: adjusted for age, sex, waist, height

Model 2a: adjusted for age, sex, waist circumference, height, smoking, physical inactivity, actual hypertension, triglyceride level, total cholesterol/HDL-cholesterol ratio

Model 2b: adjusted for model 2a covariates + HbA1c

Model 3a: adjusted for age, sex, waist circumference, height, smoking, physical inactivity, actual hypertension, parental history of diabetes, sibling history of diabetes

Model 3b: adjusted for model 3a covariates + HbA1c

[a]The outcome 'incident (pre)diabetes' includes 32 incident type 2 diabetes cases who converted directly from normoglycemia to type 2 diabetes

Supplemental Table 3 Sensitivity analysis: Multivariable adjusted logistic regression results for association between the 14 proteins (per one sex-specific SD increase) and incident prediabetes after exclusion of the 32 incident type 2 diabetic participants

Protein<br>Adjustment	Incident Prediabetes vs Non-Cases (*n*=223 vs 446)		
	OR	95% CI	*p* value
adiponectin			
Model 1	0.973	[0.803, 1.180]	0.784
Model 2a	1.051	[0.859, 1.286]	0.629
apoA-IV			
Model 1	1.065	[0.886, 1.279]	0.504
Model 2a	1.061	[0.881, 1.277]	0.534
apoC-II			
Model 1	1.082	[0.905, 1.294]	0.385
Model 2a	1.062	[0.858, 1.314]	0.580
apoC-III			
Model 1	1.056	[0.861, 1.294]	0.603
Model 2a	1.027	[0.797, 1.325]	0.836
apoE			
Model 1	1.146	[0.981, 1.338]	0.086
Model 2a	1.128	[0.955, 1.332]	0.155
CD5L			
Model 1	1.037	[0.867, 1.241]	0.691
Model 2a	1.067	[0.888, 1.283]	0.488
CRP			
Model 1	1.149	[0.955, 1.383]	0.142
Model 2a	1.099	[0.908, 1.330]	0.332
GPLD1			
Model 1	1.087	[0.900, 1.313]	0.388
Model 2a	1.101	[0.905, 1.340]	0.336
MASP			
Model 1	**1.241**	**[1.034, 1.488]**	**0.020**
Model 2a	**1.248**	**[1.036, 1.503]**	**0.020**
MBL2			
Model 1	1.013	[0.810, 1.267]	0.907
Model 2a	0.982	[0.784, 1.231]	0.876
PZP			
Model 1	1.072	[0.923, 1.245]	0.361
Model 2a	1.054	[0.905, 1.227]	0.499
RBP4			
Model 1	0.923	[0.775, 1.100]	0.372
Model 2a	0.930	[0.774, 1.118]	0.439
SHBG			
Model 1	0.975	[0.837, 1.135]	0.745
Model 2a	0.977	[0.834, 1.144]	0.775

Supplemental Table 3 *continued*

Protein<br>Adjustment	Incident Prediabetes<br>vs Non-Cases (*n*=223 vs 446)		
	OR	95% CI	*p* value
THBS1			
Model 1	1.044	[0.865, 1.260]	0.654
Model 2a	1.018	[0.840, 1.233]	0.858

Statistically significant results are printed in bold.

Model 1: adjusted for age, sex, waist, height

Model 2a: adjusted for age, sex, waist circumference, height, smoking, physical inactivity, actual hypertension, triglyceride level, total cholesterol/HDL-cholesterol ratio

Supplemental Table 4 AUC-estimates for study data as it is (not using bootstrap-validation) of selected proteins for incident type 2 diabetes and incident (pre)diabetes

Basic Prediction Model	Incident Type 2 Diabetes (*n*=783) Selected Proteins	Basic AUC (95% CI)	Extended AUC (95% CI)	Delta AUC	*p* value DeLong-Test	Incident (Pre)diabetes (*n*=701) Selected Proteins	Basic AUC (95% CI)	Extended AUC (95% CI)	Delta AUC	*p* value DeLong-Test
$GDRS_{adapted}$[a]	MASP, adiponectin, apoE	0.790 (0.751, 0.829)	0.818 (0.780, 0.856)	**0.028**	**0.011**	MASP	0.752 (0.716, 0.788)	0.756 (0.720, 0.791)	0.004	0.367
Age + sex + HbA1c	MASP, adiponectin, apoE	0.821 (0.780, 0.862)	0.841 (0.802, 0.879)	**0.019**	**0.010**	MASP, CRP	0.726 (0.687, 0.764)	0.743 (0.706, 0.780)	0.017	0.062
$GDRS_{adapted}$ + HbA1c[b]	MASP, adiponectin, apoE, PZP	0.850 (0.815, 0.886)	0.860 (0.825, 0.896)	0.010	0.123	MASP	0.781 (0.747, 0.816)	0.786 (0.752, 0.820)	0.004	0.299

AUC = area under the receiver operating characteristic curve: Basic AUC without proteins, Extended AUC with selected proteins.

Statistically significant results are printed in bold.

[a]Model 3a: $GDRS_{adapted}$ prediction variables: age, sex, waist circumference, height, smoking, physical inactivity, actual hypertension, parental history of diabetes, sibling history of diabetes

[b]Model 3b

SUPPLEMENTAL FIGURE

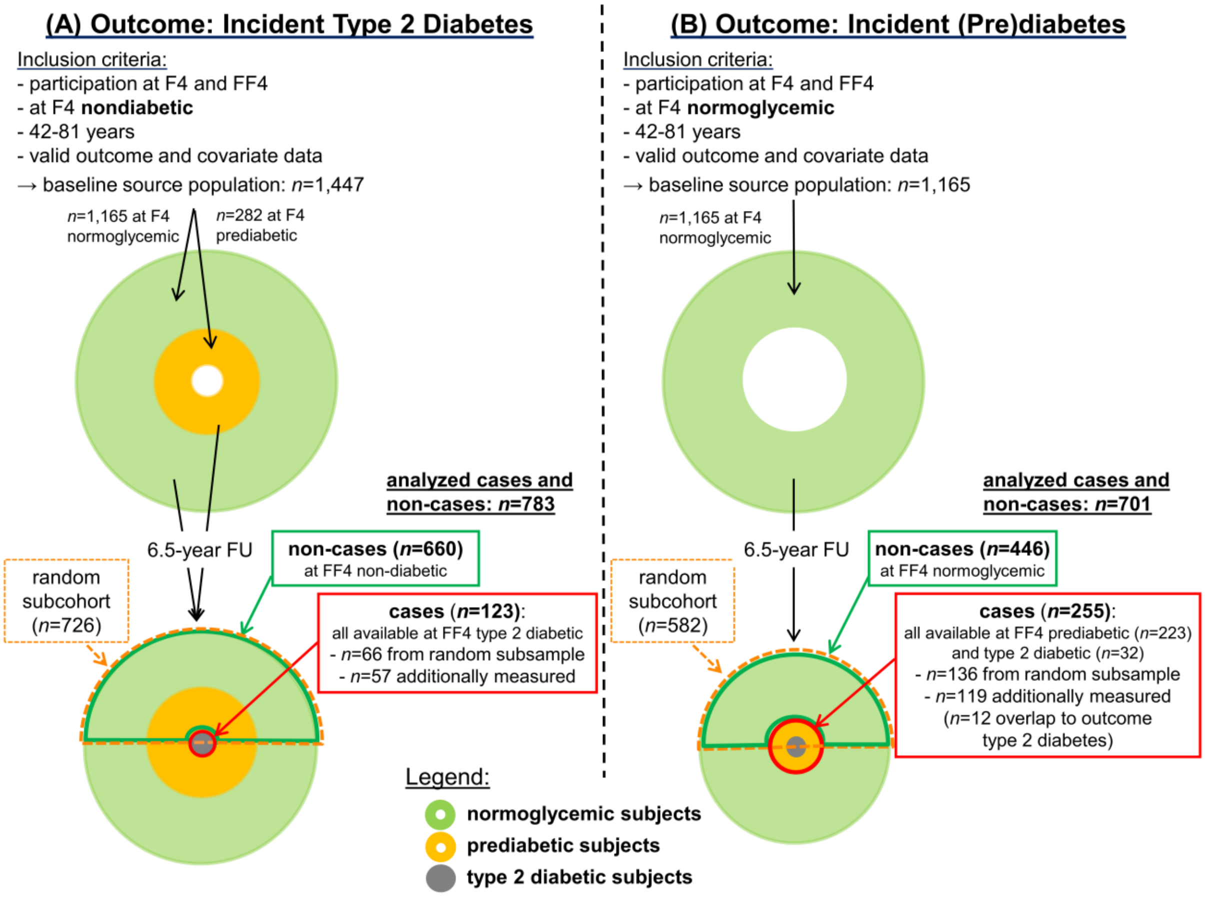

Supplemental Fig. 1 Illustration of the case-cohort design for (A) incident type 2 diabetes (left part of the figure) and (B) incident (pre)diabetes (right part of the figure). The sample sizes of the baseline source population and the analyzed participants are presented in detail.

(A): At KORA FF4 incident type 2 diabetic cases and non-cases were at KORA F4 nondiabetic (normoglycemic or prediabetic). Plasma KORA F4 samples were measured.

(B): At KORA FF4 incident (pre)diabetic cases and non-cases were at KORA F4 normoglycemic. Plasma KORA F4 samples were measured.

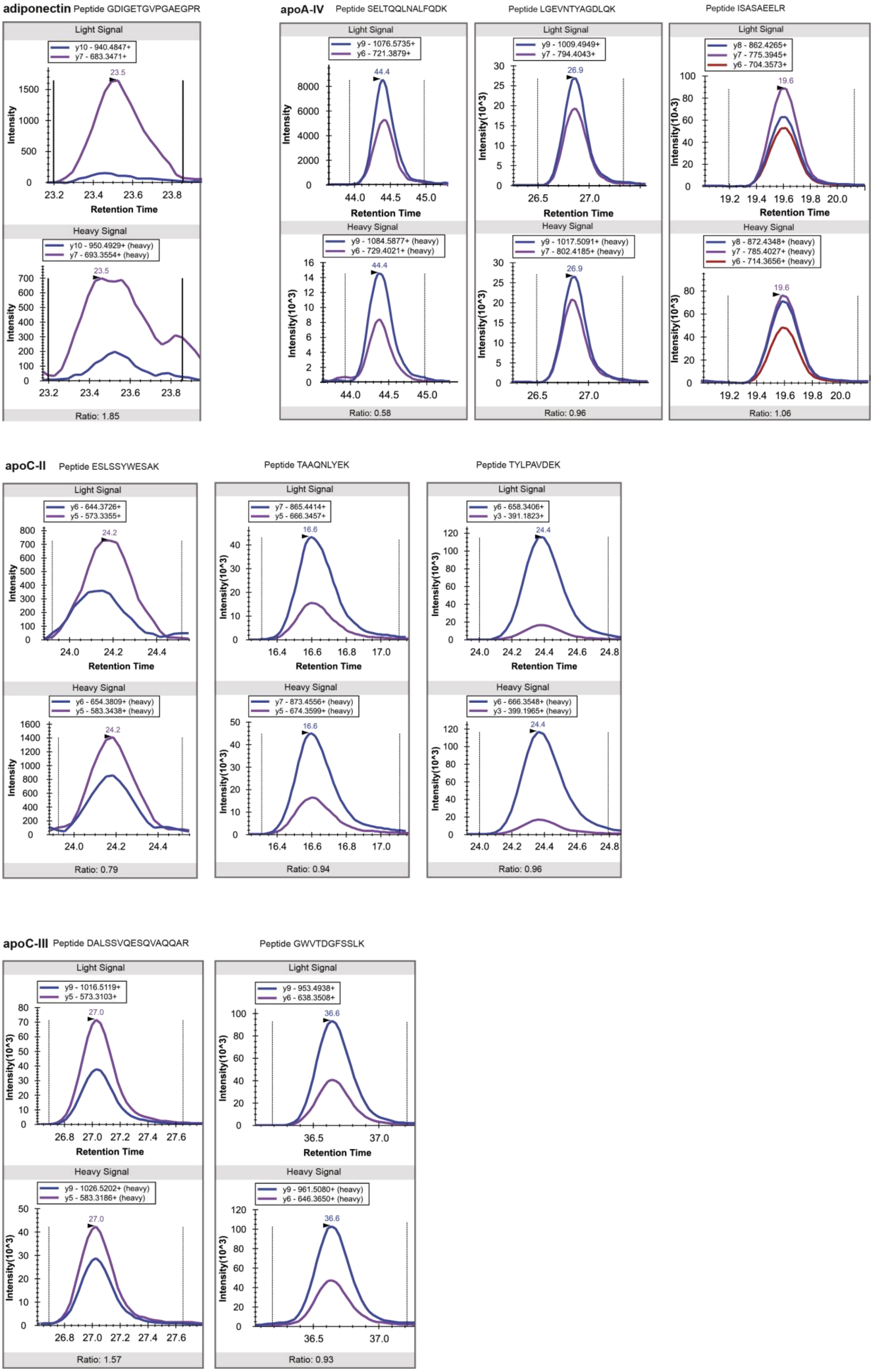

adiponectin Peptide GDIGETGVPGAEGPR
Light Signal
y10 - 940.4847+
y7 - 683.3471+
Intensity
Retention Time
Heavy Signal
y10 - 950.4929+ (heavy)
y7 - 693.3554+ (heavy)
Ratio: 1.85
apoA-IV Peptide SELTQQLNALFQDK
Light Signal
y9 - 1076.5735+
y6 - 721.3879+
Heavy Signal
y9 - 1084.5877+ (heavy)
y6 - 729.4021+ (heavy)
Intensity(10^3)
Ratio: 0.58
Peptide LGEVNTYAGDLQK
y9 - 1009.4949+
y7 - 794.4043+
y9 - 1017.5091+ (heavy)
y7 - 802.4185+ (heavy)
Ratio: 0.96
Peptide ISASAEELR
y8 - 862.4265+
y7 - 775.3945+
y6 - 704.3573+
y8 - 872.4348+ (heavy)
y7 - 785.4027+ (heavy)
y6 - 714.3656+ (heavy)
Ratio: 1.06
apoC-II Peptide ESLSSYWESAK
y6 - 644.3726+
y5 - 573.3355+
y6 - 654.3809+ (heavy)
y5 - 583.3438+ (heavy)
Ratio: 0.79
Peptide TAAQNLYEK
y7 - 865.4414+
y5 - 666.3457+
y7 - 873.4556+ (heavy)
y5 - 674.3599+ (heavy)
Ratio: 0.94
Peptide TYLPAVDEK
y6 - 658.3406+
y3 - 391.1823+
y6 - 666.3548+ (heavy)
y3 - 399.1965+ (heavy)
Ratio: 0.96
apoC-III Peptide DALSSVQESQVAQQAR
y9 - 1016.5119+
y5 - 573.3103+
y9 - 1026.5202+ (heavy)
y5 - 583.3186+ (heavy)
Ratio: 1.57
Peptide GWVTDGFSSLK
y9 - 953.4938+
y6 - 638.3508+
y9 - 961.5080+ (heavy)
y6 - 646.3650+ (heavy)
Ratio: 0.93

apoE Peptide SELEEQLTPVAEETR — Peptide AATVGSLAGQPLQER — Peptide LEEQAQQIR

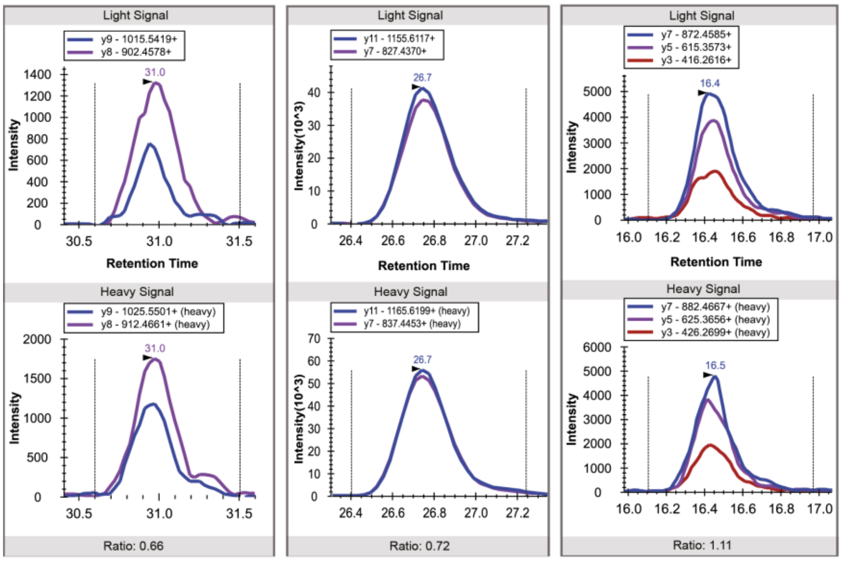

CRP Peptide ESDTSYVSLK — Peptide GYSIFSYATK

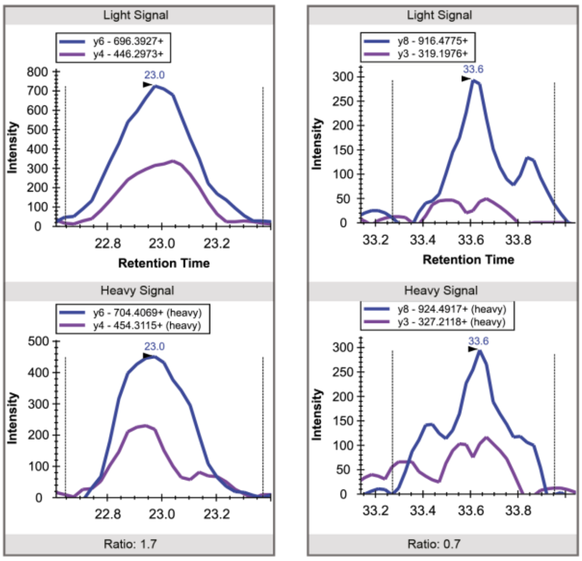

CD5L Peptide EATLQDCPSGPWGK — Peptide NTCNHDEDTWVECEDPFDLR — Peptide LVGGDNLCSGR

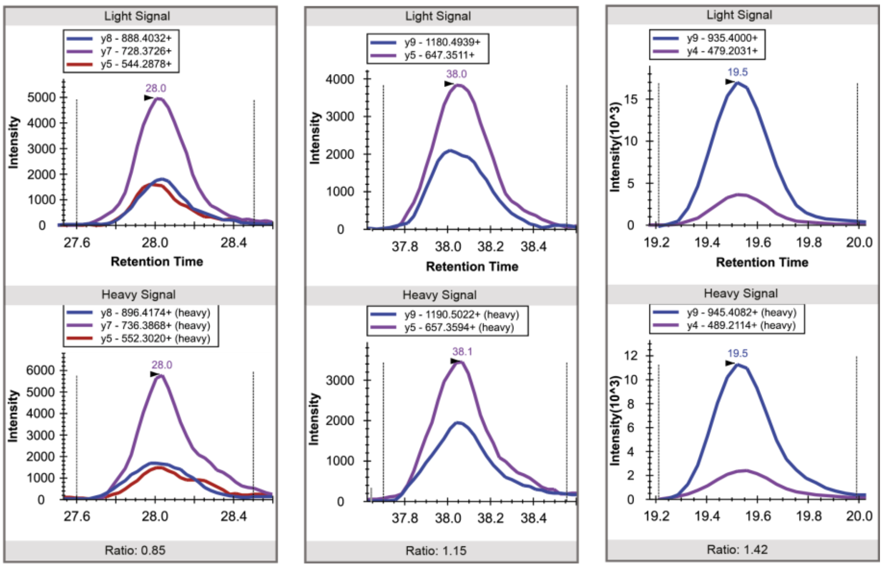

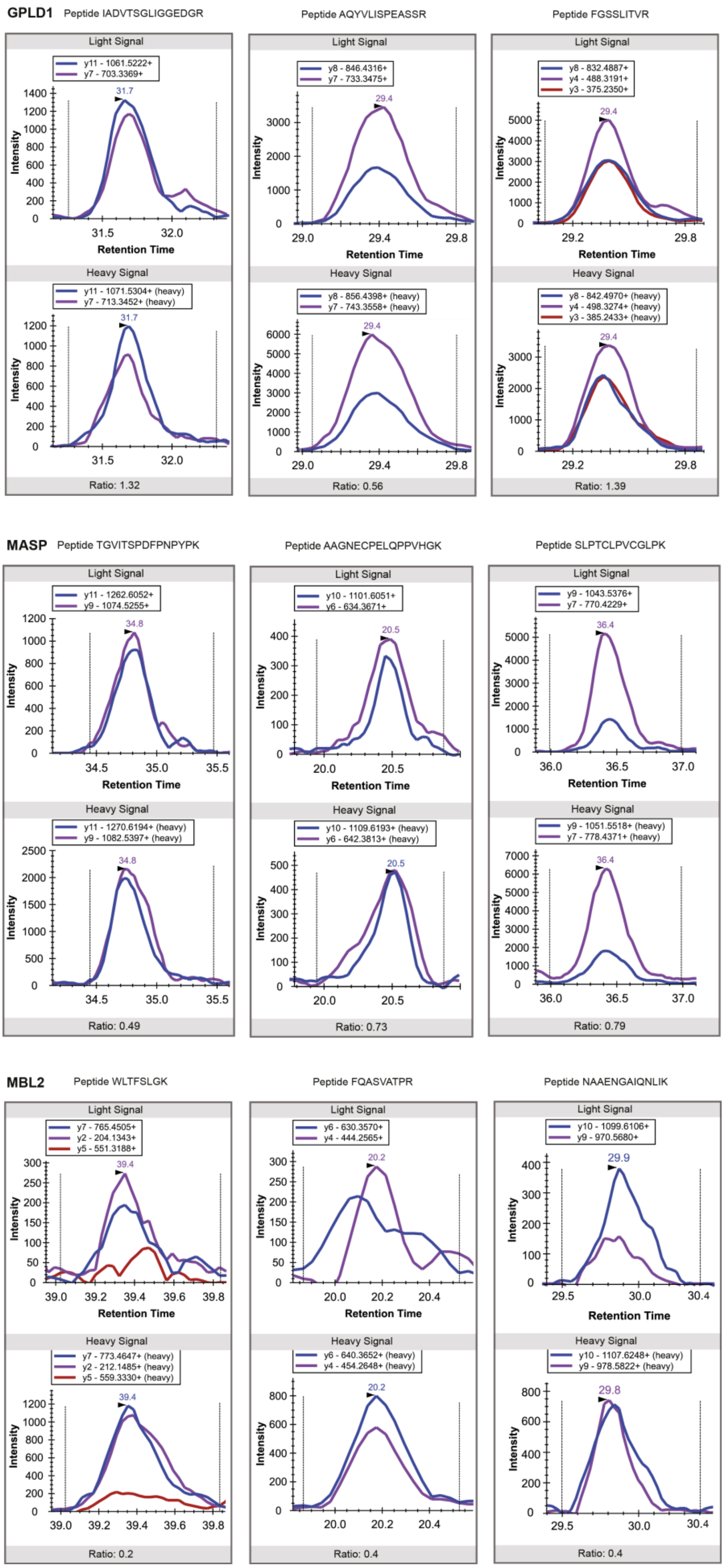
GPLD1
Peptide IADVTSGLIGGEDGR
Peptide AQYVLISPEASSR
Peptide FGSSLITVR
Light Signal
Heavy Signal
Retention Time
Intensity
Ratio: 1.32
Ratio: 0.56
Ratio: 1.39
MASP
Peptide TGVITSPDFPNPYPK
Peptide AAGNECPELQPPVHGK
Peptide SLPTCLPVCGLPK
Ratio: 0.49
Ratio: 0.73
Ratio: 0.79
MBL2
Peptide WLTFSLGK
Peptide FQASVATPR
Peptide NAAENGAIQNLIK
Ratio: 0.2
Ratio: 0.4
Ratio: 0.4

PZP Peptide TLLVEAEGIEQEK

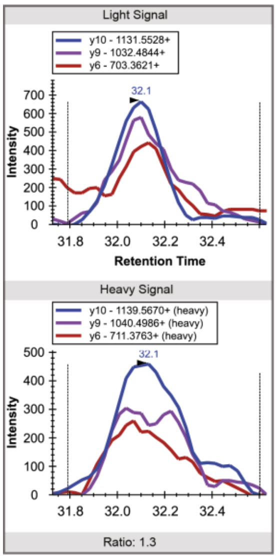

RBP4 Peptide YWGVASFLQK

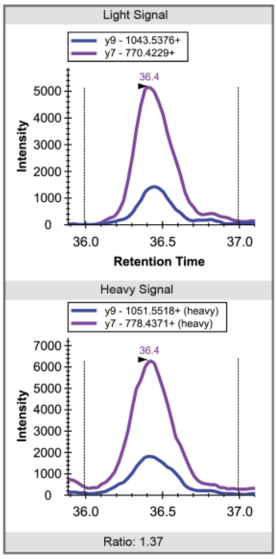

Peptide LIVHNGYCDGR

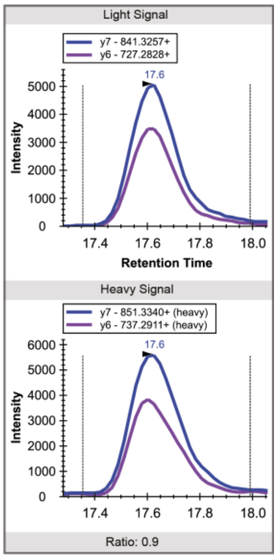

SHBG Peptide IALGGLLFPASNLR

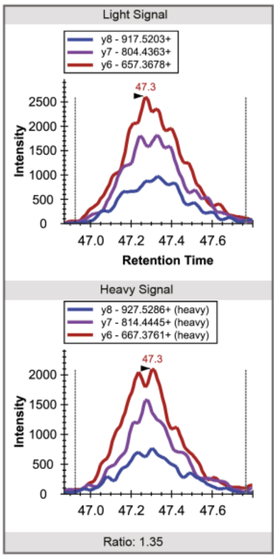

Peptide QAEISASAPTSLR

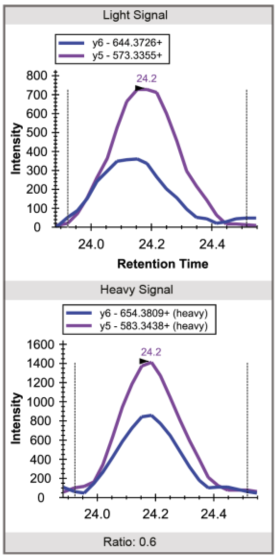

THBS1 Peptide GGVNDNFQGVLQNVR

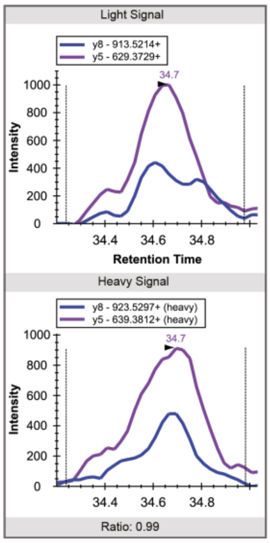

Peptide TIVTTLQDSIR

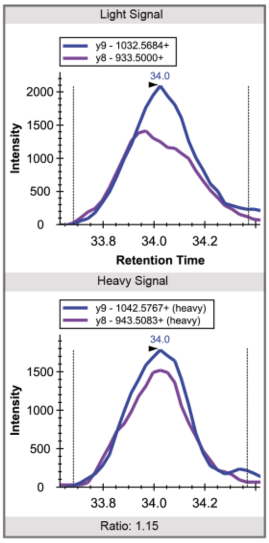

Supplemental Fig. 2 This multipanel figure displays endogenous (light) and synthetic (heavy) transitions for the 31 peptides which represent the 14 proteins quantified in this study. For example, the y10 and y7 transitions were used for quantification of the adiponectin peptide GDIGETGVPGAEGPR. Because the figure shows all used transitions, three transitions are shown in case different transitions were used in lot 1 and lot 2 measurements (e.g. for the apoA-IV peptide ISASAEELR). The actual mass-to-charge (m/z) values measured by the mass spectrometer for each transition are displayed in the small boxes above the transition curves. For example, for adiponectin the m/z value of the endogenous peptide's y10 transition is 940.4847. The m/z values of the heavy transitions are about 8 or 10 units higher compared to the light transitions, dependent on the heavy isotopes used in the purchased heavy peptides. For example, for adiponectin the m/z value of the y10 transition of the heavy peptide is 950.4929. The displayed data originate from a pooled sample used for quality control. The x-axis displays the retention time, which confirms matching of light and heavy signals. The y-axis displays an arbitrary intensity used for relative quantification. This intensity is peptide dependent and can only be compared between samples, not across peptides. The transition signals are quantified by calculation of the area under the curve (AUC), using the software Skyline. The heavy peptides were spiked in to generate light to heavy AUC ratios of roughly 1. On this basis, quantification is most exact. The ratios (displayed at the bottom of each transition panel) vary from 1 due to technical reasons. The subsequent computations yielding relative protein concentrations are depicted in suppl. fig. 3.

Depletion
and **digestion**
of KORA F4 plasma

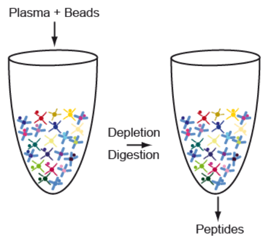

SRM-MS measurement
31 peptides representing
14 candidate proteins

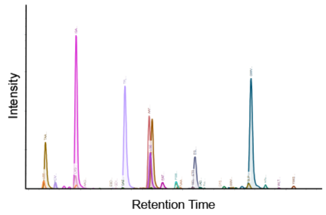

SRM-MS signal integration
of all light and heavy
transitions of all peptides

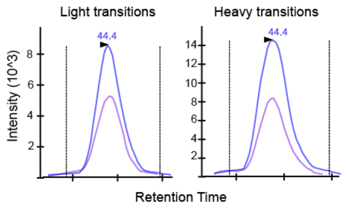

Computation of
sex standardized
relative protein
concentrations

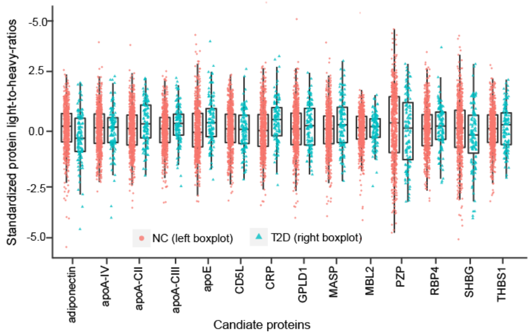

Computation of
age- and sex-adjusted
odds ratios for incident
T2D per 1 SD increased
protein level

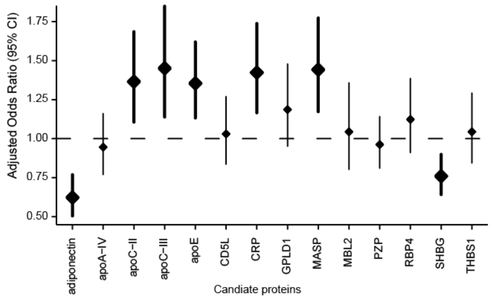

Computation of
multivariable adjusted
odds ratios for incident
T2D per 1 SD increased
protein level

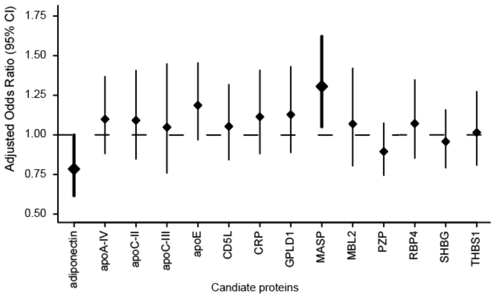

Supplemental Fig. 3 This schematic workflow displays the major SRM-MS data analysis steps, described in detail in the methods subchapters 'targeted SRM-MS protein measurements' and 'statistical analysis'. After sample preparation and mass spectrometric measurement, the signal intensities of all selected transitions of all used endogenous (light) and synthetic (heavy) peptides are recorded and quantified with the Skyline software. The transition signals of all investigated peptides are displayed in suppl. fig. 2. Panel 3 of this figure shows the signal intensities of one pooled sample exemplarily for one peptide and separately for the light and heavy transitions. The area under the curve (AUC) information from up to three transitions per peptide were exported from the Skyline software to R. Only the two transitions having the most similar information were subsequently used. Light to heavy ratios (LHRs) of the AUC-values were calculated, log_2-transformed, averaged for the transitions of each peptide and corrected for technical covariates. The peptide-level LHR information was averaged per protein and divided by the sex-specific standard deviation to yield relative protein levels. Panel 4 displays boxplots for the unadjusted relative concentrations of all investigated proteins, separately for incident type 2 diabetic cases (T2D) and non-cases (NC). Panel 5 illustrates the age- and sex-adjusted odds ratios for incident type 2 diabetes per 1 SD increased protein level. Statistically significant results in panels 5 and 6 ($p<0.05$) are displayed in bold. The multivariable adjusted panel 6 odds ratio figure is the same as shown in figure 2 of the main manuscript.
